# Four-dimensional imaging in teleorthodontics – analysis of accuracy in patients with dentofacial deformities compared with controls

**DOI:** 10.1186/s12903-025-07169-7

**Published:** 2025-10-31

**Authors:** Tim-Boyke Janssen, Jérémy Mouchoux, Bernhard Wiechens, Henning Schliephake, Philipp Meyer-Marcotty, Anja Quast

**Affiliations:** 1 Department of Orthodontics, University Medical Center Goettingen, Göttingen, Germany; 2 Department of Maxillofacial Surgery, University Medical Center Goettingen, Göttingen, Germany

**Keywords:** Telemedicine, Remote consultation, Image processing, Malocclusion

## Abstract

**Background:**

Teleorthodontics may offer benefits to both patients and providers in terms of accessibility, efficiency, and quality of care. Therefore, there is an increasing need for accurate, noninvasive monitoring technologies. The present study evaluated the accuracy of a mobile device for four-dimensional imaging in capturing dynamic facial movements and its potential application in orthodontic patients with severe dentofacial deformities.

**Methods:**

In this prospective clinical study, facial movements of 30 adult orthodontic patients (dentofacial deformity: *n* = 20, controls: *n* = 10) were captured to evaluate the accuracy of a 4D mobile camera system. Imaging was conducted via Intel RealSense D415 in three settings: (1) with high-accuracy preset, (2) with medium-density presets, and (3) with additional tracking software. The stationary system Canfield Vectra H5 served as the gold standard. Each patient was asked to perform three movements: maximum smiling, lip pursing, and cheek puffing. Accuracy was assessed by the root mean square (RMS).

**Results:**

In total, 270 4D sequences were analyzed. Overall, the RMS between the 4D sequences and the gold standard were smaller than 1 mm (*p* < .001). The (a) high-accuracy setting results in a significantly lower RMS than does the (b) medium-density setting (*p* < .001). Setting (c) resulted in higher RMS values for lip pursing and cheek puffing than setting (a) or (b). Among the movements, smiling presented the greatest RMS, whereas lip pursing and cheek puffing presented similarly small differences. Class-wise comparisons did not reveal evidence of differences.

**Conclusion:**

The present study demonstrated that the portable 4D camera system achieved sub-millimeter accuracy in patients with dentofacial deformities. Accuracy was influenced by the recording preset, whereas additional software did not improve performance. Within the limits of this pilot study, the device showed adequate recording quality to support feasibility in teleorthodontic applications for capturing facial dynamics. Future research should evaluate its usability, cost-effectiveness, and potential integration with artificial intelligence to extend clinical applications.

## Introduction

Orthodontic procedures constitute the third largest treatment category in dentistry for children and adolescents up to 20 years of age, with total expenditures for orthodontics in the United States nearly doubling from 1996 to 2016 [1, 2]. Despite this growth, the trend is not mirrored in teledentistry. Although systematic reviews have confirmed the efficacy and clinical benefits of teledentistry [3–5], its adoption in orthodontics remains limited [6]. A recent review identified only four clinical studies in teleorthodontics, focusing on patient screening, treatment planning, and monitoring [7–11]. This highlights the need for further research and application in this area.

While most orthodontic patients are aged 12–18, adults make up approximately a quarter of all orthodontic visits [12]. Treating adults is more challenging due to the lack of growth, especially in severe dentofacial deformities where orthodontics alone is often insufficient. In such cases, an interdisciplinary orthodontic-surgical approach in which orthodontists align the dental arches and craniomaxillofacial surgeons correct jaw relations is necessary [13, 14]. The goal of this therapy is to achieve normal occlusion, acceptable soft tissue, harmonic skeletal proportions, and dentofacial aesthetics [15]. The mean duration of the total treatment is 27.8 months, with scheduled appointments every four to eight weeks [16], which means that high patient adherence is needed. An approach to improve patients’ flexibility and convenience is teleorthodontics. Implementing a mobile device to monitor postsurgical recovery might help save patients’ time and increase their mental comfort and quality of care. Through digital monitoring, both orthodontists and craniomaxillofacial surgeons can see patients at the same time, which improves interdisciplinary communication.

Facial motion analysis has long been of clinical interest. Initially, movement was assessed using two-dimensional videos (2D) or static images of rest and maximal expression [17, 18], which failed to capture the continuous, muscle-driven nature of facial dynamics. Stereophotogrammetry later introduced a reliable method for three-dimensional soft tissue imaging based on triangulation of 2D images captured simultaneously by multiple cameras, achieving geometric errors below 0.2 mm [19–22]. Based on this, videostereophotogrammetry added a temporal component (fourth dimension = time) and provides the opportunity to capture dynamically changing surfaces. This offers new modalities of treatment monitoring and enables clinicians to quantify postsurgical swelling and recovery of facial mobility objectively. Moreover, it creates the opportunity to compare the muscular equilibrium of surgical patients with that of those who receive orthodontic treatment alone. However, most 4D imaging systems are stationary, expensive and thus far rarely used in the daily clinical setting. This limitation can be overcome by the use of portable, cost-effective 4D cameras. These compact systems have the further advantage that they can serve as portable monitoring systems in teleorthodontics and may cover the high demand for dental video consultation, which became obvious during the COVID-19 pandemic [23, 24]. Most handheld devices use infrared (IR) sensor-based imaging technology. This offers greater independence from ambient lighting conditions and enables real-time depth data capture, making it suitable for diverse clinical environments. Furthermore, IR sensor-based systems are often smaller and more compact, making them easier to integrate into portable devices [25].

These technological advancements raise the question of whether portable 4D cameras can deliver sufficient accuracy for clinical use. In orthodontics and orthognathic surgery, where regular monitoring of facial dynamics is essential, mobile systems could offer a practical alternative to stationary setups. However, despite their theoretical advantages, the clinical performance and limitations of such mobile devices remain largely unexplored.

Therefore, the objectives of this study were (1) to assess the overall accuracy of a portable IR sensor-based 4D camera in a clinical setting, (2) to examine the impact of presets and recording software on this accuracy, and (3) to investigate whether severe dentofacial deformities and everyday expressions influence accuracy.

## Materials and methods

###  Population

For this prospective clinical study, 30 adults were recruited from the pool of orthodontic patients at the Department of Orthodontics, University Medical Center Goettingen. The participants were selected on the basis of their Wits appraisal and categorized into three skeletal classes: Class I (Wits = 0 ± 2 mm, indication for orthodontic treatment), Class II (Wits > + 2 mm, severe skeletal deformity and indication for orthodontic-surgical treatment), and Class III (Wits < −2 mm, severe skeletal deformity and indication for orthodontic-surgical treatment). Each class comprised 10 participants. The inclusion criteria were orthodontic therapy with a fixed appliance and pronounced malocclusion. Patients with cleft lip and/or palate, craniofacial syndromes, impaired facial motion, previous orthognathic surgery, or beards were excluded from the study. As this was a prospective clinical pilot study, no formal sample size calculation was performed. The aim was to explore the feasibility and accuracy of a mobile 4D imaging system rather than to test a specific hypothesis. A sample of 30 participants was chosen based on feasibility and comparability with similar studies, providing sufficient preliminary data to guide future research.

For patients with skeletal Class II and III, imaging was conducted six weeks before the surgical procedure. Images of patients with skeletal Class I were captured during a routine orthodontic appointment.

In accordance with the Declaration of Helsinki, this study was approved by the institutional ethics committee (application number 13/2/19). All participants provided written informed consent prior to data acquisition.

### Data acquisition

All video recordings and postprocessing were conducted by a single trained investigator (T.-B. J.). The portable 4D imaging system used was the Intel RealSense D415 (Intel Corp., Santa Clara, CA, USA), which is a depth camera that uses stereo vision to capture 3D videos. The camera incorporates two depth sensors, an infrared sensor, and an image sensor. These sensors record both visible and infrared light, from which 30 3D images per second are generated via a dense stereo matching algorithm. The RealSense device captures images with a color resolution of 1920 × 1080 pixels and a depth resolution of 1280 × 720 pixels. The patients were positioned at a distance of approximately 0.5 m from the camera. The focus range was adapted by adjusting the depth units to 100 μm and manually setting the disparity shift to 23 units in the advanced settings to increase the accuracy at close range.

**Fig. 1:**
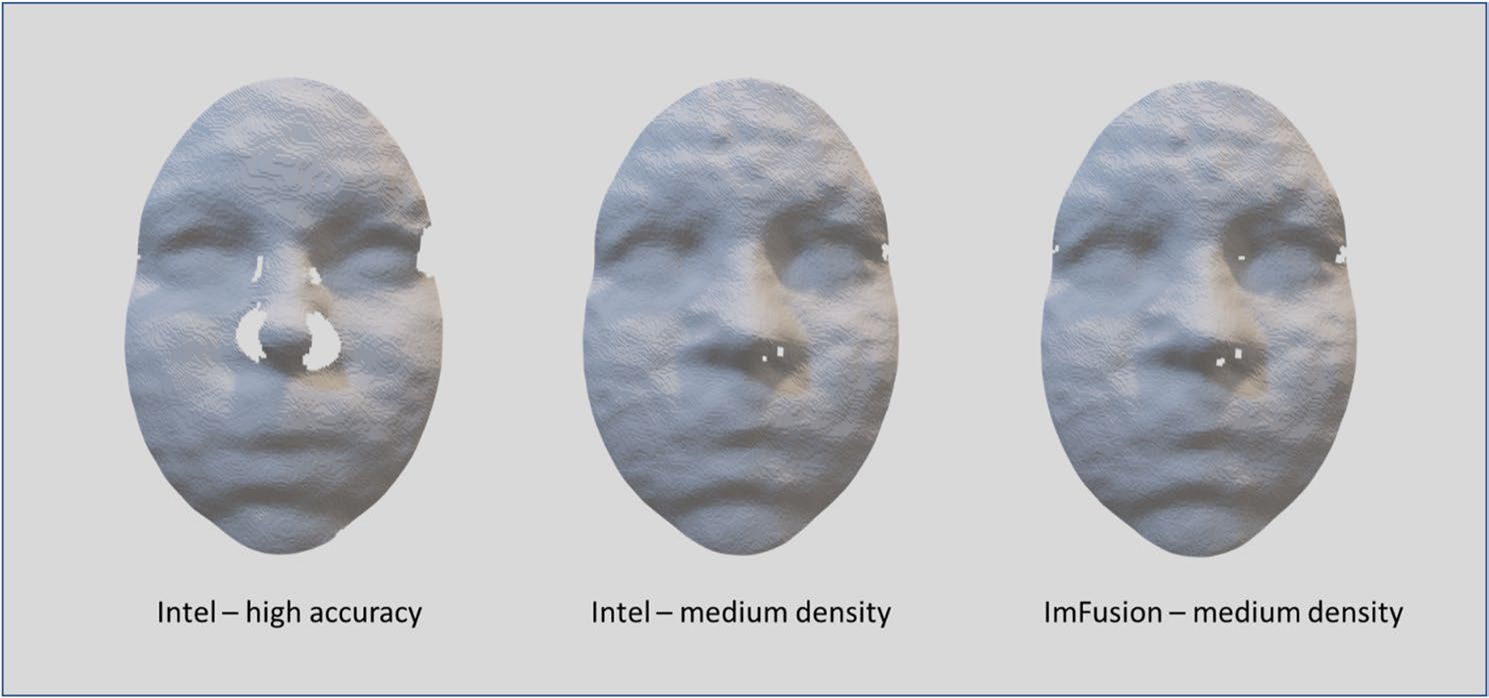
Surface reconstructions of a participant’s face recorded with different camera presets and software. Examples are shown for the Intel D415 4D camera using **a** the high-accuracy preset, **b** the medium-density preset, and** c** the Recfusion software (ImFusion, Munich, Germany) based on the medium-density preset. The comparison illustrates how recording quality and completeness vary depending on preset configuration and processing software

The D415 camera offers several preset configurations that adjust the camera's performance to suit specific needs. In this study, two different presettings were used (see Figure1):

- High Accuracy Mode: This setting maximizes the precision of depth measurements. It is particularly useful in controlled lighting conditions where capturing fine details is critical but at the expense of density.
- Medium Density Mode: This mode offers a balance between accuracy and data density. It is designed to provide a dense depth map without compromising the accuracy too much.
  In addition to Intel’s proprietary program,
- Recfusion recording software (Imfusion, Munich, Germany) was used.
  These supplementary recordings were designed to demonstrate the extent to which recording inaccuracies were attributable not only to the camera’s hardware but also to the software. The recording with the Recfusion software was based on the medium density preset.

The Canfield Vectra H5 imaging system (Canfield Scientific, Parsippany, NJ, USA) was set as the gold standard for accuracy testing of the mobile camera. The Vectra system is designed for medical 3D recordings in the head and neck area with a field of view (FOV) of 360° and consists of five cameras. It provides detailed surface geometry capture, making it an excellent tool for baseline comparisons. Several studies have proven the accuracy and reproducibility of Vectra systems [26].

Prior to each recording session, both camera systems were calibrated according to the manufacturers’ specifications. Calibration of the Intel RealSense D415 required the use of the Dynamic Calibrator software (Intel, Santa Clara, USA) in combination with a printed calibration pattern. The Canfield Vectra H5 system was calibrated using a manufacturer-provided reference panel designed specifically for the device.

Each patient was asked to perform three facial movements: (1) maximum smiling, (2) lip pursing, and (3) cheek puffing. These movements have been verified for their reproducibility in previous studies and are state-of-the-art for evaluating facial dynamics [27]. Before the actual data were captured, the patients performed each movement several times.

For data acquisition, the recording by RealSense D415 was started, and the participants were instructed to move from a neutral facial expression to their maximum capability of smiling, lip pursing or cheek puffing. Concurrently, the patients were captured by the Vectra H5 camera system as the peak movement was reached. Each movement was recorded in three settings: (a) with high accuracy preset, (b) with medium density preset, and (c) with the additional Recfusion software.

###  Data processing

Each 4D video was recorded at a speed of 30 frames per second. To assess the accuracy of the videos, the 3D image that was recorded two frames after the flash appeared in the 4D video was selected for further analysis. The reason for selecting this minimally delayed frame was the brief overexposure of the Intel sensors by the flash of the Canfield Vectra system. A recording speed of 30 frames per second corresponds to a delay of 0.066 s.

**Fig. 2:**
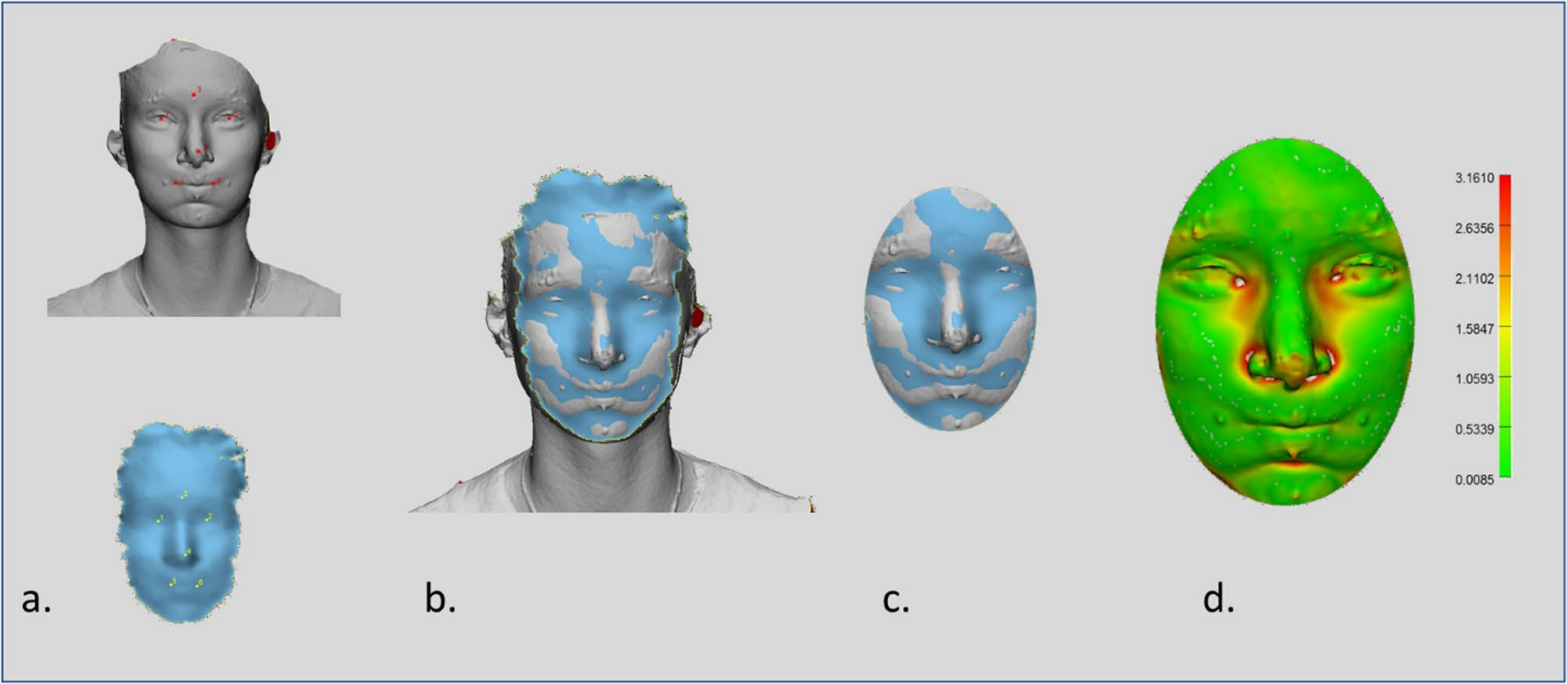
Alignment and error analysis of 4D recordings with the reference 3D Vectra images. **a** Example of a exported Vectra scan as goldstandard and the corresponding 4D recording with manually placed landmarks for initial n-point registration. **b** Alignment of 4D and Vectra datasets after global registration, refining the fit by iterative distance minimization. **c **Oval region of interest cropped to include only mimically relevant facial areas.** d** Color-coded distance map showing root mean square (RMS) deviations between the two datasets, with the scale bar indicating distance in millimeters. Processing was performed in 3-matic software (Materialise, Belgium)

Subsequent processing of the data was conducted via 3-matic software (Materialise, Belgium; Fig. 2). The 3D exports from the 4D recordings were aligned with the corresponding Vectra images by n-point registration, using manually selected landmarks on both datasets (right and left cheilion, pronasale, mid-eyes, and mid-forehead). A subsequent global registration refined the alignment through algorithms that progressively minimized the distance between the datasets. To compare only mimically relevant areas, an oval section of the face was selected. To assess the accuracy of the portable 4D camera, the root mean square (RMS) between the 4D sequences and the 3D Vectra images was calculated.

### Statistical analysis

Descriptive statistics and analyses were performed via SPSS (v. 29, IBM, New York, USA). The overall accuracy of the portable camera, i.e., the RMS between the 4D sequences and the 3D Vectra images, was compared to a hypothetical mean of 1 mm via a one-sample t test. The threshold of 1 mm was selected as a technical benchmarking reference because previous studies on multimodal image registration (e.g., superimposition of 3D facial photographs with CT) reported deviations in the range of 0.8–1.0 mm [28, 29]. We therefore used 1 mm as a pragmatic reference value for comparing the performance of the portable 4D system, while explicitly acknowledging that clinical acceptability is task-dependent. An analysis of variance (ANOVA) with repeated measures was employed to assess the RMS values for differences between the following factors: (1) the skeletal class, (2) the type of movement (smiling, lip pursing, and cheek puffing), and (3) the recording modality (setting a) presetting high accuracy, setting b) presetting medium density and setting c) additional Recfusion software). The movement and the recording modality were defined as within-subject factors, whereas the skeletal class served as a between factor. The α-level was set at 0.05, and Greenhouse–Geisser estimates were reported for the main and interaction effects. All post hoc tests were Bonferroni corrected.

## Results

### Study sample

In total, 270 4D sequences (30 participants × 3 camera settings × 3 movements) of 30 participants were analyzed. Detailed information on the clinical and demographic characteristics of the participants is displayed in Table 1.

**Table 1:** Demographic and clinical profile of participants included in the analysis

Study sample	*n* = 30
Gender (female/male)	20/10
Age (years)	M = 29.17 SD = 9.34
Wits (mm)	M = −0.40 SD = 5.19
Skeletal class I	*n* = 10
Gender (female, male)	7/3
Age (years)	M = 30.40 SD = 11.97
Wits (mm)	M = −0.51 SD = 0.81
Skeletal class II	*n* = 10
Gender (female, male)	8/2
Age (years)	M = 30.50 SD = 9.34
Wits (mm)	M = 5.53 SD = 2.21
Skeletal class III	*n* = 10
Gender (female, male)	5/5
Age (years)	M = 26.60 SD = 6.29
Wits (mm)	M = −6.23 SD = 2.12

### Overall accuracy and effect of the factors skeletal class, type of movement and recording software

The overall discrepancy between the 4D sequences and the 3D Vectra images was smaller than 1 mm (RMS: M = 0.89, SD = 0.07, (*p* <.001)). Both the recording modality and the type of movement had a significant main effect on the RMS, with high effect sizes, whereas there was no significant main effect for skeletal class (Table 2). No interaction effect of the recording modality and the type of movement was observed (F(2.96, 79.98) = 0.605, *p* =.611).

**Table 2:** Results of the analysis of variance (ANOVA) with repeated measures for the main effects of skeletal class, type of movement and recording modality. Displayed are F-values, degrees of freedom, p-values, and effect sizes (partial η²). Significant main effects were found for recording modality and type of movement, but not for skeletal class

Factor	*p*-value	partial η²
Skeletal Class F(2, 27) = 2.88	0.073	0.18
Type of movement F(1.5, 40.64) = 0.605	< 0.001	0.49
Recording modality F(1.45, 39.03) = 90.24	< 0.001	0.77

###  Accuracy depending on the recording setting

Recordings with high accuracy presented the lowest deviation from the gold standard (RMS: M = 0.82, SD = 0.06). In contrast, recordings with setting b) medium density presented significantly greater RMS values (M = 0.91, SD = 0.07, *p* <.001). Setting c) additional Recfusion software resulted in the highest deviations (RMS: M = 0.94, SD = 0.08, *p* <.001). A detailed comparison of the recording modality revealed that within the Intel software, the high-accuracy preset presented a significantly lower RMS for all movements than did the medium-density preset, with a consistently high effect size (see Table 3). Conversely, setting c) demonstrated a significantly greater RMS for lip pursing and cheek puffing than did setting b), albeit with only low–to-medium effect sizes.

**Table 3:** Pairwise comparisons of RMS differences between recording settings for each facial movement (smile, lip purse, cheek puff). Mean differences (M), standard deviations (SD), significance levels (p-values), and effect sizes (Cohen’s d) are shown. Across all movements, the high-accuracy preset yielded significantly lower RMS values than the medium-density preset, while the recfusion setting showed the highest deviations

Difference in RMS: setting (a) high accuracy vs. setting (b) medium density				
	***M***	***SD***	***p*** **-value**	**Cohen’s** ***d***
Smile	−0.11	0.08	< 0.001	1.32
Lip purse	−0.8	0.08	< 0.001	1.02
Cheek puff	−0.09	0.07	< 0.001	1.27
Difference in RMS: setting b) medium density vs. setting c) additional software				
	***M***	***SD***	***p*** **-value**	**Cohen’s** ***d***
Smile	0.02	0.02	0.295	0.20
Lip purse	0.03	0.01	0.024	0.43
Cheek puff	0.03	0.06	0.004	0.57

### Accuracy depending on the type of movement

The 4D sequences with the movement smile demonstrated the greatest deviation from the gold standard (RMS: M = 0.94, SD = 0.11) compared with recordings with the movement lip purse (RMS: M = 0.86, SD = 0.06, *p* =.001) or cheek puff (RMS: M = 0.87, SD = 0.06, *p* <.001). This difference was obvious across all the recording modalities (Table 4).

The RMS difference between the movements of the lip purse and cheek puff was not significant in any of the recording modalities.

**Table 4:** Pairwise comparisons of RMS differences between facial movements (smile, lip purse, cheek puff) within each recording setting a)–c). Mean differences (M), standard deviations (SD), significance levels (p-values), and effect sizes (Cohen’s d) are shown. Smiling consistently produced the largest RMS deviations compared with lip pursing and cheek puffing across all recording modalities

Difference in RMS: smile vs. lip purse				
	***M***	***SD***	***p*** **-value**	**Cohen’s** ***d***
Setting a) high accuracy	0.07	0.08	< 0.001	0.93
Setting b) medium density	0.08	0.13	< 0.001	0.78
Setting c) additional software	0.09	0.93	< 0.001	0.97
Difference in RMS: smile vs. cheek puff				
	***M***	***SD***	***p*** **-value**	**Cohen’s** ***d***
Setting a) high accuracy	0.06	0.08	< 0.001	0.79
Setting b) medium density	0.08	0.13	0.003	0.59
Setting c) additional software	0.07	0.10	0.001	0.65
Difference in RMS: lip purse vs. cheek puff				
	***M***	***SD***	***p*** **-value**	**Cohen’s** ***d***
Setting a) high accuracy	−0.01	0.06	0.375	0.17
Setting b) medium density	−0.02	0.07	0.147	0.27
Setting c) additional software	−0.02	0.07	0.089	0.32

## Discussion

The present study demonstrated that facial movements can be captured by a portable 4D camera system with a 1 mm accuracy, making it an interesting tool for teleorthodontics. However, the type of movement and software presets used might affect accuracy, which should be considered when evaluating soft tissue dynamics. The RMS values for smiling were significantly greater than those for lip pursing and cheek puffing. This discrepancy can be explained by the fact that the camera’s closely spaced sensors struggle with objects nearly perpendicular to the camera plane, such as the lip curvature during smiling. Reduced accuracy in the corners of the mouth and the curves of the lips was expected [30]. This highlights the need for specific calibration of the camera system on the basis of the type of movement being assessed. For example, smile dynamics, which have higher RMS values due to the perpendicular orientation of the lips and mouth corners, could benefit from alternative camera angles or additional frames to increase spatial data reliability during teleconsultations.

In accordance with our expectations, the high-accuracy preset results in significantly lower RMS values than presets with medium density. This is probably because the high accuracy preset uses only data points with very high confidence. However, the 0.09 mm difference might be clinically negligible in most cases. Consequently, the 4D sequences using medium density presets with Intel’s native software appear to offer a balanced solution, providing sufficient accuracy while maintaining manageable file sizes. This balance is crucial in teleorthodontic applications, where data transmission speeds and storage limitations could impact the clinical workflow. A recent study also showed that reducing frame rates to 30 or even 15 fps does not significantly compromise accuracy, while substantially decreasing data volume, which may further support clinical feasibility of 4D facial imaging in resource-limited settings [31]. However, while the difference in RMS values between high and medium density presets is minimal, this minor discrepancy might become clinically relevant in cases requiring precise measurement of soft tissue changes over time, such as in surgical orthodontics or for monitoring facial asymmetry. For such applications, high accuracy settings could still be preferable.

Interestingly, Intel software outperforms Recfusion for two of the three movement types. This requires further exploration into the algorithms underlying each software’s data processing. Differences in data interpolation methods or point-tracking algorithms could influence outcomes and suggest that not all software solutions are equally suitable for dynamic, multiplane movement capture. Future studies could benefit from a detailed analysis of these algorithms to determine how software-specific processing affects the precision of 4D facial movement data.

The deviation range observed with the mobile 4D camera system should be considered within the broader landscape of clinically accepted imaging tolerances. The camera’s performance aligns well with other routinely used imaging techniques, which often display deviations within a range of 0.8–1.0 mm, as noted in studies overlaying 3D facial photographs with CT data [28, 29]. The comparison with stationary 4D camera systems, which achieve deviations as low as 0.2 mm [32], highlights a key trade-off: while stationary systems offer unparalleled accuracy, their lack of portability limits their use in teleorthodontics and patient monitoring outside of clinic settings. Unlike stationary systems that are typically used under controlled lighting, portable devices may be affected by changing ambient light conditions [33]. Although the D415 uses infrared sensors, some influence of lighting on measurement accuracy cannot be entirely excluded. However, portable systems such as Intel D415, despite having slightly greater deviations, present a valuable alternative for routine assessment and follow-up in a teleorthodontics framework. The minor increase in deviation does not significantly compromise clinical decision-making, especially for applications where tracking relative changes over time is more crucial than capturing absolute positional data.

The present findings suggest that careful adjustments to recording parameters, such as disparity shift and depth units, may significantly increase the accuracy of portable 3D cameras in teleorthodontics. The observed reduction in RMS deviation (0.89 ± 0.07 mm) relative to the results reported by Harkel and colleagues for the Intel RealSense F200 (1.48 ± 0.28 mm) and D415 (0.97 ± 0.07 mm) [30, 34] demonstrates the importance of fine-tuning camera settings to meet the specific requirements of facial imaging. Although the authors used Intel software, they did not specify recording modes or presets. However, this information is essential for implementing mobile cameras in clinical settings. The exclusion of postprocessing filters in the current study provides a more unaltered assessment of the camera’s baseline performance, offering a more realistic view of the device’s capabilities and limitations. Employing postprocessing techniques to reduce RMS may have introduced a layer of artificial smoothing, potentially masking certain inaccuracies that could arise in a real-world teleorthodontic setting. Another inherent limitation of the system is the absence of facial texture, which means that the captured models do not convey the real face unless combined with photographic textures or supplementary 2D images.

One limitation of the study is the small sample consisting of symmetric patients only. Facial asymmetries, especially in the nose and mouth region, might affect the quality of the recordings, as areas perpendicular to the camera with undercuts significantly reduce recording accuracy. Consequently, the generalizability of our findings is restricted to relatively symmetric patients under controlled conditions. Future studies should therefore include larger and more heterogeneous cohorts, explicitly incorporating asymmetric cases, to confirm and extend the present results. However, the accuracy of the portable camera for symmetric patients with severe dentofacial deformities suggests that, under optimal conditions, these devices can serve as a practical, low-cost solution in teleorthodontics even though medical certification is pending. The simplicity and portability of the 4D camera system make it especially appealing for monitoring postsurgical outcomes and complex orthodontic cases remotely. For example, in orthodontic-surgical patients, the ability to assess real-time changes in facial symmetry, monitor postsurgical swelling, and evaluate recovery in facial motion without in-person visits could improve patient management and reduce unnecessary travel, particularly for those in rural or underserved areas. This approach may save time, costs, and resources while improving healthcare access [35]. In conjunction with smartphone applications, AI, and intraoral dental scans, the integration of portable 4D imaging can create a robust teleorthodontic platform [36].

Furthermore, AI is expected to play an increasingly important role in teleorthodontics. For example, recent studies have discussed its potential to automate facial analysis and support diagnostic workflows [37] as well as to redefine the tasks of dental assistants and nurses in AI-assisted remote care [38]. While these aspects were beyond the scope of our pilot study, they represent important future directions for integrating portable 4D imaging into comprehensive teleorthodontic systems.

## Conclusion

The validation of the Intel RealSense D415 camera system demonstrated its potential as a reliable tool for capturing dynamic facial movements in orthodontic patients with dentofacial deformities. The RMSs between the 4D sequences and the gold standard were smaller than 1 mm, suggesting sufficient accuracy for clinical settings. The high-accuracy setting resulted in a significantly lower RMS than did the medium-density setting. The highest RMS values were found for lip pursing and cheek puffing in the setting with additional software. While dentofacial deformities had no impact on the accuracy of the 4D sequences, the type of movement and the recording modality significantly affected the RMS value. Therefore, the portable 4D camera system presents a cost-effective, practical solution for dynamic facial movement analysis in orthodontics, with significant potential for teleorthodontics and in-office applications when the correct presets are chosen. Future research should focus on integrating portable 4D recording systems with artificial intelligence to automate data processing and analysis, further increasing the clinical utility of these devices.

## Data Availability

The data that support the findings of this study are not publicly available because they contain information that could compromise the privacy of research participants but are available from the corresponding author, AQ, upon reasonable request.
